# Responses of Growth Performance and Proinflammatory Cytokines Expression to Fish Oil Supplementation in Lactation Sows' and/or Weaned Piglets' Diets

**DOI:** 10.1155/2013/905918

**Published:** 2013-09-01

**Authors:** Jie Luo, Feiruo Huang, Chenglin Xiao, Zhengfeng Fang, Jian Peng, Siwen Jiang

**Affiliations:** ^1^Department of Animal Nutrition and Feed Science, College of Animal Science and Technology, Huazhong Agricultural University, Wuhan 430070, China; ^2^Key Laboratory of Swine Breeding and Genetics of Agricultural Ministry, College of Animal Science and Technology, Huazhong Agricultural University, Wuhan 430070, China

## Abstract

The study was conducted to investigate whether dietary fish oil could influence growth of piglets via regulating the expression of proinflammatory cytokines. A split-plot experimental design was used with sow diet effect in the main plots and differing piglet diet effect in the subplot. The results showed that suckling piglets from fish oil fed dams grew rapidly (*P* < 0.05) than control. It was also observed that these piglets had higher ADG, feed intake, and final body weight (*P* < 0.05) during postweaning than those piglets from lard fed dams. Furthermore, there was a significant decrease (*P* < 0.01) in the expression of interleukin 6 and tumor necrosis factor-**α** in *longissimus dorsi* muscle. In contrast, there was a tendency (*P* < 0.10) towards lower ADG and higher feed : gain in weaned piglets receiving fish oil compared with those receiving lard. Meanwhile, splenic proinflammatory cytokines expression was increased (*P* < 0.01) in piglets receiving fish oil during postweaning period. The results suggested that 7% fish oil addition to sows' diets alleviated inflammatory response via decreasing the proinflammatory cytokines expression in skeletal muscle and accelerated piglet growth. However, 7% fish oil addition to weaned piglets' diets might decrease piglet growth via increasing splenic proinflammatory cytokines expression.

## 1. Introduction

The growth rate of piglet is most rapid during the early postnatal stage, and it is very important to the subsequent growth, final body weight, and marketing time of pigs. Many researches revealed that fish oil could affect young piglets' growth [1–5]. It was reported that 3~5% of fish oil addition to sows' diets was beneficial to suckling piglet growth [1, 5]. However, lactation sow diet supplemented with 8~10% fish oil increased piglets' preweaning morbidity and decreased sows milk production and litter weight gain [2, 6]. Dietary supplementation with 2~3% fish oil promoted weaned piglets growth [3], whereas body weight and feed intake of weaned piglets fed 5% or more fish oil were lower versus corn oil [4].

Previous studies have demonstrated that proinflammatory cytokines could increase muscle protein degradation, reduce muscle protein synthesis, divert nutrients to the synthesis of components in the immune system, and suppress animal growth [7–9]. Fish oil has anti-inflammatory and immunomodulatory effect. Studies in animal models and in human subjects generally reported a decreased production of proinflammatory cytokines in immune cell in peripheral blood and spleen after fish oil supplementation [10–12], and the anti-inflammatory effect has also been shown for suckling piglets of fish oil fed sows [13]. Further study revealed that the immunomodulatory effect was caused by the (n-3) polyunsaturated fatty acids ((n-3) PUFA), especially, eicosapentaenoic acid (EPA, C20:5 (n-3)) and docosahexaenoic acid (DHA, C22:6 (n-3)) in the fish oil [14]. Remarkably, (n-3) PUFA could decrease the proinflammatory cytokines (interleukin 1 (IL-1), IL-6, and tumor necrosis factor-*α* (TNF-*α*)) expression and secretion in peripheral immune cells and suppress animals' inflammatory response [5, 11, 15]. (n-3) PUFA could attenuate the growth inhibition effect by reducing the production of proinflammatory cytokines in several species [4, 16, 17]. Our previous study found that intake of n-3 PUFA leads to significant decreases in the expressions of proinflammatory cytokine genes in loin muscle and spleen, which may stimulate growth in growing-finishing barrows [18]. However, several studies have shown that high level of fish oil (30% or more) supplementation in mice diet increased TNF-*α* production in splenocytes [19, 20]. In the present study, we hypothesized that the inconsistent performance of piglets that ingested different levels of fish oil mentioned before was due to the different response of proinflammatory cytokines expression to (n-3) PUFA supplementation. Thus, piglets receiving lard directly from the postweaning diet or indirectly from the lactation sows' diets were used as the control. The aim of the present study was to investigate the effect of dietary supplementation of 7% fish oil in lactation sows' and/or weaned piglets' diets on growth performance of piglets and expression of proinflammatory cytokines including IL-1*β*, IL-6, and TNF-*α* in skeletal muscle and spleen.

## 2. Materials and Methods

### 2.1. Animals and Diets

This trial was carried out in accordance with Huazhong Agricultural University Animal Care and Use Committee guidelines. A split-plot experimental design was used with sow diet effect (L) in the main plots and differing piglet diet effect (PW) in the subplot. Landrace × large white sows (*n* = 20) at 10 d before parturition were assigned to 1 of 2 groups matched for parity and body weight. The test group (T) received a diet supplemented with 7% fish oil, while control group (C) received an isoenergetic, isonitrogenous, and isolipidic diet with 7% lard oil. Fish oil and lard were purchased from China National Cereals, Oils & Foodstuffs Corp. (COFCO Limited). The oil and fat were for food or feed-quality grade. And 500 mg/kg ethoxyquin was added to fish oil and lard as antioxidative. The two diets were formulated to meet NRC requirements of nutrient standards for lactation sow [21]. All diets were prepared weekly to keep them fresh. Two sows in the test group gave birth to less than 3 piglets and thus were dropped from the study. Upon farrowing, sows were fed their treatment diet twice daily (0800 and 1600 h). Sows were initially fed 2.0 kg, and this was increased daily by ~0.5 kg of feed until d 4 postpartum, depending on sows' feed consumption and recovery after parturition. After d 4 postpartum, sows had free access to their diets until weaning. The composition of the sow diets is shown in Table 1. Two sows of the fish oil fed group gave birth to less than 3 piglets and thus were rejected. At farrowing, litters were equalized within dietary treatments to the same number of piglets *per* litter (*≈*10). Prestarter feed was freely available to the nursing piglets from 7 d of age until weaning.

At 28 d of age, 56 piglets, 28 piglets (half females and half castrated males) *per* group of sows, were moved to cages and reared in nursery room. All piglets remained in the same treatment group defined by their dam; they were then subdivided into two groups of 14 piglets each (one female and one castrated male as a replication) such that a total of 4 experimental groups obtained: CC (control sows-control piglets), CT (control sows-treated piglets), TC (treated sows-control piglets), and TT (treated sows-treated piglets). Piglets from CT and TT groups were fed a starter diet supplemented with 7% fish oil, and an isoenergetic, isonitrogenous, and isolipidic diet supplemented with 7% lard was used as the starter diet fed to CC and TC piglets. The two diets were formulated to meet NRC requirements of nutrient standards for piglet [21]. The composition of the piglet diets is shown in Table 1. Weaned piglets were fed experimental diets from 35 d to 70 d after farrowing. The feed intake of piglets was recorded daily. The body weight of piglets was measured at d 0, 21, 35, and 70 postnatal. The piglets' average daily gain (ADG) during 0~21 d and 35–70 d were calculated as body weight gain/days.

### 2.2. Collection of Milk and Tissue Samples

At d 21 of lactation, 30~40 mL of milk was collected from the functional glands of each sow after injection of 2 mL of oxytocin. The milk samples were immediately frozen at −20°C for later analysis. The milk samples were analyzed to determine fatty acid composition.

Sixteen piglets (4 *per* treatment, half females and half castrated males) were slaughtered at the end of the experiment. The pigs were deprived of feed for 12 h before humanely slaughter via electrically stun and exsanguination. The samples of the *longissimus dorsi* muscle were collected between the tenth and last ribs, and spleen samples were collected at cacumen. All the collected samples were snap frozen in liquid nitrogen and stored at −80°C for subsequent RNA isolation.

### 2.3. Fatty Acids Analysis

Fatty acids composition of milk (10 mL), diets (1 g), and diced muscle (2 g) were analyzed by gas chromatography. Lipids were extracted by chloroform: methanol (1 : 1) as described by Folch et al. [22]. Fatty acid methyl ester was prepared for gas chromatography determination using KOH/methanol (0.4 mol/L) [23]. 

The CP-3800 gas chromatography (Varian, Inc., USA) equipped with a 1177 injector, a flame ionization detector, and a capillary chromatographic column CPSil88 (Varian, Inc., USA) (50 m × 0.25 mm × 0.20 *μ*m) for fatty acid methyl ester was used in this experiment. The injector and detector temperatures were kept at 250°C and 270°C, respectively. Nitrogen was used as carrier gas with a flow rate of 1.0 mL/min, and the split ratio was 1 : 100. The column temperature was programmed as follows: 100°C at first, increased to 200°C (5°C/min), and held constant for 5 min; then, the temperature was increased to 225°C (2°C/min) and kept constant for 2 min. The total analysis time was 39.5 min. The fatty acids were identified by comparing the retention times of the peaks with those of known standards (Sigma Chemical Co., St. Louis, Mo). Response factors for the fatty acids were calculated using the same standard mixtures plus an internal standard [24].

Fatty acid results are presented as g/100 g fatty acids. Saturated fatty acids are the sum of C14:0, C16:0, and C18:0. The monounsaturated fatty acids are the sum of C16:1 (n-7) and C18:1 (n-9). The (n-3) PUFA are the sum of C18:3 (n-3), C20:5 (n-3), C22:5 (n-3), and C22:6 (n-3). The (n-6) PUFA are the sum of C18:2 (n-6) and C20:4 (n-6). The sum of the PUFA was calculated as the sum of (n-3) PUFA and (n-6) PUFA.

### 2.4. Reverse Transcription Polymerase Chain Reaction (RT-PCR)

Total RNA was extracted using the TRIzol regent (Invitrogen Corp., Carlsbad, CA, USA) according to the manufacturer's specifications. The RNA samples were quantified spectrophotometrically at 260 and 280 nm. A two-step semiquantitative RT-PCR method was used to measure gene expression [25]. Oligo-(dT)_20*n*_ (Toyobo, Osaka, Japan) was used as the primer in the first step of cDNA synthesis. Reverse transcription reaction solution (20 *μ*L) consisted of 2 *μ*g of total RNA, 100 U of MMLV (Moloney Murine Leukemia Virus) reverse transcriptase (Toyobo, Osaka, Japan), 20 U of an RNAse inhibitor (Toyobo, Osaka, Japan), 0.5 mmol/L of deoxyribonucleotide triphosphates (dNTP) (Toyobo, Osaka, Japan), and 0.5 *μ*L oligo-dT primers. The cDNA stock was stored at −20°C. The yield of cDNA was measured according to the PCR signal generated from the internal standard housekeeping gene, **β*-actin,* which was amplified from 25 to 35 cycles starting with 0.5 *μ*L of the cDNA solution. The volume of each cDNA pool was adjusted to give the same exponential-phase PCR signal intensity for **β*-actin* after 25 cycles [26].

Relative RT-PCR [27] was performed to measure gene expression of *IL-1*β**, *IL-6*, and *TNF-*α** of *longissimus dorsi* muscle and spleen. Primer sequences and optimal PCR annealing temperatures (*t*_*a*_) are listed in Table 2. PCR was performed on a 2720 Thermal Cycler (Applied Biosystems, USA). The PCR program began with a 94°C denaturation for 5 min, followed by 25~35 cycles denatured at 94°C for 30 s, annealing for 30 s, and extension for 30 s at 72°C, with a final extension at 72°C for 10 min. The linear amplification range for each gene was tested on the adjusted cDNA. The optimal cycle number was then considered to be two cycles lower than the highest cycle of linearity. The PCR products were electrophoresed on a 1.5% agarose gel and stained with ethidium bromide (10 *μ*g/mL). The gel images were digitally captured with G:BOX (Syngene, Cambridge, UK) and densitometry values were measured using the Gene Tool software (Syngene, Cambridge, UK). RT-PCR values are presented as a ratio of the specified gene signal in the selected linear amplification cycle divided by the **β*-actin* signal. Data for each replicate represented the mean of three individual RT-PCRs.

### 2.5. Statistics

Experimental animals were assigned to different treatments in a completely randomized design. Pen was the experimental unit. Statistical analysis of the data was performed with the ANOVA procedure of SAS8.01 [28], and the residual error was used to test the main effect of dietary treatments. The multiple comparisons were preceded with DUNCAN procedure. Data were presented as means ± SEM. Growth performance and gene expression data of postweaning piglets were analyzed as 2 × 2 randomized ANOVA with lactation (L) and postweaning diet period (PW) as effects. The correlation between ADG and expressions of proinflammatory cytokine genes was performed with the CORR procedure of SAS8.01 [28]. Means were considered statistically different at *P* < 0.05.

## 3. Results

### 3.1. Fatty Acids Composition of Diets and Milk

Fatty acid composition of sow and piglet diets is shown in Table 3. Fatty acid composition of sow milk at d 21 of lactation is shown in Table 4. Fish oil diets and lard diets had the same levels of lipids, while the (n-3) PUFA were nearly 8~9 times higher in fish oil diets as compared to lard diets. Compared with lard oil, fish oil administration increased the concentration of PEA, DHA and total (n-3) PUFA in sows' milk (*P* < 0.01), whereas, the EPA, DHA, and total (n-3) PUFA contents in milk from fish oil fed sows were lower than those in fish oil added diets which fed to piglets during postweaning period (7.00 g/100 g total fatty acid versus 10.28 g/100 g total fatty acid). As a result, piglets from fish oil fed dams received fewer amount of (n-3) PUFA than weaned piglets fed 7% fish oil diet (0.41% versus 1.00%).

### 3.2. Fatty Acids Composition in *Longissimus Dorsi *Muscle

Fatty acid composition of *longissimus dorsi *muscle is shown in Table 5. The saturated fatty acids (C14:0, C16:0, C18:0, and C20:0), C20:4n-6, and n-6 PUFA contents in *longissimus dorsi *muscle of postweaning piglets from fish oil fed dams were significantly lower (*P* < 0.05) than those of piglets from control sows. The C20:1n-9 (0.78% versus 0.62%) and C22:5n-3 (0.84% versus 0.2%) contents were significantly increased (*P* < 0.05) in piglets from sows compared with those from control sows.

 The C16:0, C20:0, and SFA contents in *longissimus dorsi *muscle of piglets receiving fish oil were significantly decreased (*P* < 0.05) compared with those receiving control diets during the postweaning period. The C20:5n-3 (1.39% versus 0.13%), C22:5n-3 (0.56% versus 0.47%), C22:6n-3 (1.38% versus 0.34%), and total n-3 PUFA (4.38% versus 1.3%) contents in *longissimus dorsi *muscle of piglets receiving fish oil were significantly increased (*P* < 0.01) more than those receiving control diets during the postweaning period.

### 3.3. Growth Performance of Piglets

Growth performance of suckling piglets is shown in Table 6. The body weights at birth and litter weights at delivery were not different (*P* > 0.05) between groups. However, the average daily gain (ADG) of suckling piglets was significantly (*P* < 0.05) higher in the fish oil group than in the control group. 

The growth performance of weaned piglets was also significantly (*P* < 0.05) affected by sow diet effect (L) (Table 7). The final body weights (21.29 kg versus 18.79 kg) of postweaning piglets from fish oil fed dams were significantly higher (*P* < 0.05) than those of piglets from control sows. The ADG (325 g/d versus 265 g/d) and average daily feed intake (ADFI) (615 g/d versus 510 g/d) were also significantly increased (*P* < 0.05) in piglets from sows compared with those from control sows, whereas the feed conversion rate (1.91 versus 1.96) was not different (*P* > 0.05) between groups. 

The growth performance of weaned piglets was not significantly (*P* > 0.05) affected by piglet diet effect. However, there was a tendency (*P* < 0.10) towards lower ADG (270 g/d versus 321 g/d) and higher feed : gain (2.00 versus 1.87) in piglets receiving fish oil compared with those receiving control diets during the postweaning period, but the ADFI (534 g/d versus 591 g/d) and final body weights (19.18 kg versus 20.90 kg) were not significantly (*P* > 0.05) different between groups. Additionally, final body weight, ADG, and ADFI were significantly higher in group TC than those in group CT (*P* < 0.05).

### 3.4. Proinflammatory Cytokines Gene Expression in *Longissimus Dorsi* Muscle and Spleen

**Figure 1:**
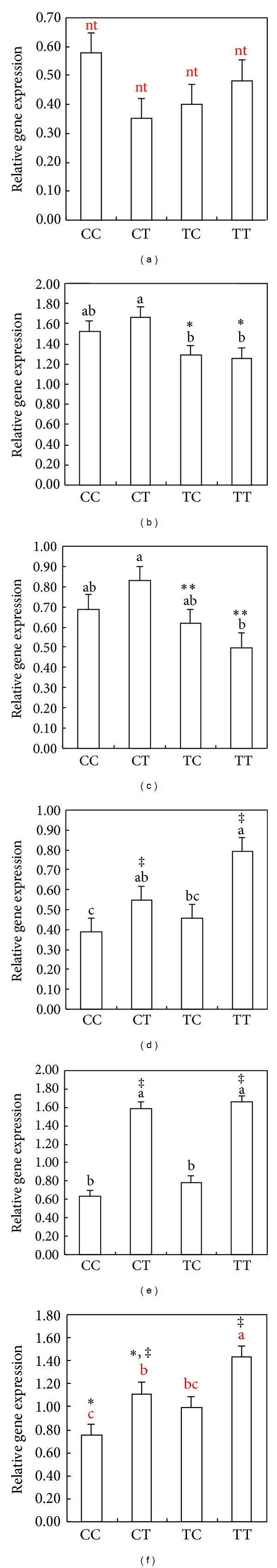
Relative *longissimus dorsi muscle* and spleen cytokines expression of weaned piglets born to fish oil-treated or control sows, and piglets fed diets with and without fish oil supplementation during the postweaning period. (a), (b), and (c) were *longissimus dorsi muscle* cytokines expression; (d), (e), and (f) were spleen cytokines expression. (a) and (d): *IL-1*β** expression; (b) and (e): *IL-6* expression; (c) and (f): *TNF-*α** expression. Gene signals were normalized to the expression of the housekeeping gene **β*-actin* optical density signal. CC: all period fed lard diet; CT: postweaning period fed fish oil diet; TC: lactation period fed fish oil diet; TT: all period fed fish oil diet. Data shown are mean values ± SEM, *n* = 4. ^∗,∗∗^: means lactation decrease effect with *P* < 0.05, *P* < 0.01; ^†,‡^: means postweaning increase effect with *P* < 0.05, *P* < 0.01; ^a,b^: means with different letters are significantly different (*P* < 0.05) among groups; ^nt^: means have no significant difference.

Figure 1 shows the results of cytokines expression in* longissimus dorsi* muscle and spleen of weaned piglets in C-C, C-T, T-C, and T-T groups. *IL-6* (Figure 1(b)) and *TNF-*α** (Figure 1(c)) expression in *longissimus dorsi* muscle was significantly (*P* < 0.05) decreased in weaned piglets from fish oil fed dams compared with those of piglets from lard fed dams, while the *IL-1*β** expression in *longissimus dorsi* muscle (Figure 1(a)) was not different (*P* > 0.05). Additionally, the *IL-1*β** and *IL-6* (Figures 1(d) and 1(e)) expression in spleen was not different (*P* > 0.05). However, postweaning piglets from fish oil fed dams had higher (*P* < 0.05) *TNF-*α** (Figure 1(f)) expression in spleen.

Proinflammatory cytokines expression of weaned piglets in *longissimus dorsi* was not affected by postweaning fish oil supplementation (PW) or L × PW interaction (*P* > 0.05) (Figures 1(a), 1(b), and 1(c)). However, postweaning fish oil supplementation resulted in significantly increased (*P* < 0.01) spleen *IL-1*β**, *IL-6*, and *TNF-*α** mRNA abundances (Figures 1(d), 1(e), and 1(f)).

### 3.5. The Correlation between ADG and Expressions of Proinflammatory Cytokine Genes

The mRNA expressions for proinflammatory cytokines in groups CC, CT, TC, and TT were pooled and compared with the ADG of the corresponding slaughtered piglets in order to evaluate a possible correlation between expressions of proinflammatory cytokines and growth performance. The correlation coefficients between ADG and expressions of proinflammatory cytokine genes are shown in Table 8. Proinflammatory cytokines' expressions in *longissimus dorsi* muscleor in spleen were negatively correlated with piglets' growth. There were statistically significant negative correlations between muscular *IL-1*β** expression (*r* = −0.7692, *P* < 0.01) or splenic *IL-1*β** expression (*r* = −0.7595, *P* < 0.01) levels and ADG.

## 4. Discussion

Daily supplementary low level of cod liver oil (50 mL/d, approximately 1%~2%) to sows from day 107 of gestation until weaning did not affect weight gain and overall morbidity of piglets in the study by Taugbøl et al. [29]. It was suggested that 1.75% (17.5 g/kg of diet) of fish oil to pregnant diet and 3.5% (35 g/kg of diet) of fish oil to lactation diet for sows improved growth of their progeny [1]. The previous results showed that the body weight and ADG of suckling piglets at 21 d postnatal were increased by 7% (70 g/kg of diet) fish oil supplementation to the late gestation and lactation sows' diets, which agreed with other researchers [1, 30]. We also found that weaned piglets from fish oil fed dams had a higher growth rate than those from lard oil fed dams during the d 35 to 70 postnatal period. These results revealed that 7% of fish oil supplemented to sows' diets could promote the growth performance of their progeny, and this effect even lasted during the postweaning period. 

Interestingly, high level of fish oil supplementation was not beneficial for piglets' growth. It was found that 8% (80 g/kg of diet) fish oil addition to sow diet during late gestation and lactation period decreased litter weight gain of suckling piglets, compared with 8% animal fat (6). Similarly, 10% (100 g/kg of diet) fish oil addition to lactation sow diet resulted in increased piglets' preweaning morbidity and decreased sows milk production (2). We also found the same trend in the present study. The ADG and feed conversion rate (FCR) of postweaning piglets were tended to decrease after 7% of fish oil feeding. The results were in general agreement with another study showing that weaned piglets receiving 5% (50 g/kg of diet) menhaden fish oil + 1% (10 g/kg of diet) corn oil during the d 24 to 36 postnatal period had a lower body weight gain and ADFI than those fed 6% corn oil [4].

The three proinflammatory cytokines IL-1, IL-6, and TNF-*α* could induce great metabolic changes [31]. The cytokines appear to be primarily derived from macrophage-rich tissues, such as the liver and spleen, and various myelomonocytic cells as well. However, it was reported that cytokines are also produced by cells not traditionally considered to be part of the immune system such as adipocytes and myofibers [32, 33], which are effective sources and targets of cytokines [34, 35]. Proinflammatory cytokines mediate “reprogramming” of metabolism and shift the partitioning of dietary nutrients away from skeletal muscle accretion toward metabolic responses that support the immune system [7, 31]. Skeletal muscle as the biggest body tissue, amounting for 40~45% of the total body mass, is also the most quickly growing tissues during early postnatal period except for bone and nervous system. Collectively, these data suggest that the autocrine/paracrine actions of cytokines are of potential importance in muscle and thus in postnatal animal growth.

There was a paucity of data pertaining to the cytokines expression after (n-3) PUFA administration in skeletal muscle. Previous study in our laboratory showed that during normal physiological processes feeding linseed diet (rich in *α*-linolenic acid) suppressed the expression of proinflammatory cytokines in *longissimus *muscle of growing-finishing barrows and decreased serum level of TNF-*α* from 0.073 to 0.052 ng/mL during normal physiological condition. These results showed that the appropriate reduction of serum TNF-*α* levels that ranged from 0.073 to 0.052 ng/mL might be beneficial to increase the longissimus muscle mass under normal physiological condition [18]. EPA and DHA were considered to be more potent in regulating immune function [14]. Fish oil has been reported to decrease production of proinflammatory cytokines because of the high content of EPA and DHA [10–12]. The *IL-6* and *TNF-*α** expression was significantly suppressed in muscle of weaned piglets from fish oil fed dams in the present study. We also found negative correlation between ADG and proinflammatory cytokines expression. Thus, suppression of cytokines expression in muscle of piglets may positively reduce the inflammatory response in skeletal muscle, attenuate the negative effect of the potent proinflammatory cytokines, and promote piglet growth.

Remarkably, it was not always consistent with the result which moderates fish oil supplementation decreased production of proinflammatory cytokines. The study in rodents suggested that mice fed as high as 30% (300 g/kg of diet) of fish oil for 4~6 weeks increased the TNF-*α* syntheses in splenocytes [19]. Barber et al. [20] also reported that TNF-*α* secretion was increased in splenocytes separated from 4% of EPA (>30% fish oil according to our data) fed mice after stimulation with LPS. We also found that all of the three proinflammatory cytokines expressions were significantly increased in spleen after 7% of fish oil feeding during post-weaning. High production of proinflammatory cytokines in spleen, the biggest immune organ, may increase proinflammatory cytokines levels of the whole body, suppress skeletal muscle protein accretion, and impact animal growth [7, 9, 36].

The results suggested that administration of 7% fish oil to lactation sows significantly increased the growth rate of their progeny during postweaning period. However, the addition of 7% fish oil to diets of postweaning piglets was likely to decrease the growth rate and FCR of piglets. The contrary effects of 7% fish oil supplementation to sows' or weaned piglets' diets may be due to the different content of (n-3) PUFA that the piglets received. The current results demonstrated that the EPA, DHA, and total (n-3) PUFA contents of milk were lower than those of piglets' diets (2.71% versus 5.90%, 2.53% versus 2.94%, and 7.00% versus 10.28%, resp.). As a result of the lower level of lipid content in milk compared with that in the diet, piglets only received ~40% of (n-3) PUFA from milk relative to that ingested from fish oil diets directly. Our previous study found that intake of n-3PUFA could increase the n-3PUFA content in *longissimus dorsi *muscle of growing-finishing pigs [37]. In the current experiment, C22:5n-3 contents in *longissimus dorsi *muscle of postweaning piglets from fish oil fed dams were increased (0.84% versus 0.2%) more than those of piglets from control sows. However, the C20:5n-3 (1.39% versus 0.13%), C22:5n-3 (0.56% versus 0.47%), C22:6n-3 (1.38% versus 0.34%), and total n-3 PUFA (4.38% versus 1.3%) contents in *longissimus dorsi *muscle of piglets receiving fish oil were increased more than those of piglets receiving control diets during the postweaning period. These results revealed that n-3PUFA contents in the muscle were higher in piglets fed fish oil directly than that in weaned piglets from fish oil fed dams. It was further observed that the* IL-6* and *TNF-*α** expression either in the muscle or in spleen was lower in weaned piglets from fish oil fed dams than that in piglets fed fish oil directly. It could be concluded that moderate (n-3) PUFA intake was beneficial to piglets' growth by decreasing proinflammatory cytokines production and suppressing inflammatory response in skeletal muscle. However, high intake of (n-3) PUFA may promote splenic proinflammatory cytokines production and impact animal growth consequently.

In conclusion, fish oil might regulate piglet growth through modulating proinflammatory cytokines production in body tissues. Appropriate levels of fish oil supplementation to sows' diets may increase (n-3) PUFA content in milk and (n-3) PUFA ingestion of their progenies, decrease the proinflammatory cytokines expression and their unfavorable effects on skeletal muscle, and thus promote growth of piglets. However, high levels of fish oil supplementation to postweaning piglets' diets may increase splenic proinflammatory cytokines expression and thus negatively impact the growth of weaned piglets. Given that the limited replicate number of slaughtered animals in the present study may be not enough to generate a definitive conclusion, further investigation with more number of samples is required to determine the appropriate fish oil supplementation level, duration, and the precise mechanisms by which long chain (n-3) PUFA affect piglets' immunity and growth.

## Figures and Tables

**Table 1:** Ingredients and composition of sow and piglet diets (%).

Ingredient	Sow diet		Piglet diet		Nutrients	Sow diet		Piglet diet	
	C	T	C	T		C	T	C	T
Corn	57.5	56.5	48	48	DE (Mcal/kg)^3^	3.45	3.44	3.36	3.35
Wheat bran	7	8	4	4	Crude protein	17.28	17.32	19.38	19.38
Dried whey	0	0	8	8	Calcium	0.80	0.79	0.79	0.79
Lard oil^1^	7	0	7	0	Total phosphorus	0.65	0.63	0.65	0.65
Fish oil^1^	0	7	0	7	Available phosphorus^4^	0.42	0.42	0.39	0.38
Fish meal	3	3	5	5	Lys^4^	1.03	1.03	1.25	1.25
Soybean meal	21.5	21.5	24	24	Met + Cys^4^	0.61	0.61	0.77	0.77
Sodium chloride	0.35	0.35	0.35	0.35					
Calcium carbonate	0.8	0.8	0.5	0.5					
Dicalcium phosphate	1.15	1.15	1.05	1.05					
L-Lysine HCl	0.20	0.20	0.25	0.25					
Methionine	0	0	0.1	0.1					
Vitamin mineral premix	1.5^2^	1.5^2^	1.75^3^	1.75^3^					

^1^The control diet contains 70 g/kg lard oil and test group diet contains 70 g/kg fish oil; 500 mg/kg ethoxyquin was added to oil as anti-oxidative.

^2^Provided following per kg of diet. Cu: 5 mg; Fe: 80 mg; Zn: 50 mg; Mn: 20 mg; I: 0.14 mg; Se: 0.15 mg; V_A_: 2,000 IU; V_D3_: 200 IU; V_E_: 44 IU; V_k3_: 0.5 mg; V_B1_: 1 mg; V_B2_: 3.75 mg; V_B12_: 0.015 mg; biotin: 0.2 mg; folic acid: 1.3 mg; niacin: 10 mg; pantothenic acid: 12 mg.

^3^Provided following per kg of diet. Cu: 6 mg; Fe: 100 mg; Zn: 100 mg; Mn: 4 mg; I: 0.14 mg; Se: 0.3 mg; V_A_: 11,000 IU; V_D3_: 1,100 IU; V_E_: 44 IU; V_k3_: 0.5 mg; V_B1_: 1 mg; V_B2_: 3.5 mg; V_B6_: 1.5 mg; biotin: 0.05 mg; folic acid: 0.3 mg; niacin: 15 mg; pantothenic acid: 10 mg.

^4^By calculation.

**Table 2:** Oligonucleotide polymerase chain reaction primers.

Gene^1^	Accession no.	Primer source	Primer sequences (5′ → 3′)	Orientation	Product size, bp	*t*_*a*_^2^ (°C)
*IL-1*β**	M86725	Pig	ATTCGAGTCTGCCCTGTA	Forward	147	54
			TCTGGGTATGGCTTTCCT	Reverse		
*IL-6 *	M80258	Pig	GCATTCCCTCCTCTGGTC	Forward	93	58
			ATAGTGTCCTAACGCTCAT	Reverse		
*TNF-*α**	AY572787	Pig	CTCCCTCTTTGTCTCCTCC	Forward	77	54
			GCATTGGCATACCCACTCT	Reverse		
*β-actin *	SSU07786	Pig	GGACTTCGAGCAGGAGATGG	Forward	233	—
			GCACCGTGTTGGCGTAGAGG	Reverse		

^1^ *IL-1*β**: interleukin 1*β*, *IL-6*: interleukin 6, *TNF-*α**: tumor necrosis factor-*α*.

^2^t_a_: optimal PCR annealing temperature.

**Table 3:** Lipid concentration and fatty acid composition of sow and piglet diets^1^.

	Sow diet		Piglet diet	
	C	T	C	T
Lipid, g/100 g diet	11.57	11.32	9.72	9.69
Fatty acid composition, g/100 g total fatty acid				
C14:0	1.13	4.37	1.38	4.56
C16:0	23.32	22.29	24.07	22.21
C18:0	11.77	4.92	9.53	4.02
∑SFA^2^	36.22	31.58	34.98	30.78
C16:1 (n-7)	1.72	6.14	1.73	6.22
C18:1 (n-9)	35.48	26.49	31.79	22.90
∑MUFA^2^	37.20	32.63	33.52	29.12
C18:2 (n-6)	20.09	14.00	20.76	15.80
C20:4 (n-6)	0.20	0.30	0.31	0.14
∑(n-6) PUFA^2^	20.30	14.30	21.07	15.94
C18:3 (n-3)	0.92	0.97	1.05	0.97
C20:5 (n-3)	0.13	6.55	0.04	5.90
C22:5 (n-3)	0.10	0.62	0.06	0.47
C22:6 (n-3)	0.22	3.87	0.05	2.94
∑(n-3) PUFA^2^	1.37	12.01	1.20	10.28
∑PUFA^2^	21.67	26.31	22.27	26.22

^1^Fatty acids are designated by the number of carbon atoms followed by the number of double bonds. The position of the first double bond relative to the methyl (n) end of the molecule is also indicated.

^2^SFA: saturated fatty acids, MUFA: monounsaturated fatty acids, PUFA: polyunsaturated fatty acids; ∑SFA: the sum of C14:0, C16:0, and C18:0; ∑MUFA: the sum of C16:1 (n-7) and C18:1 (n-9); ∑(n-6) PUFA: the sum of C18:2 (n-6) and C20:4 (n-6); ∑(n-3) PUFA: the sum of C18:3 (n-3), C20:5 (n-3), C22:5 (n-3), and C22:6 (n-3); ∑PUFA: the sum of ∑(n-6) PUFA and ∑(n-3) PUFA.

**Table 4:** Lipid concentration and fatty acid composition of milk of sows fed with and without fish oil^1,2^.

	C	T	SEM	*P*-value
Lipid, g/100 g milk solid	5.69	5.81	0.27	0.767
Fatty acid composition, g/100 g total fatty acid				
C14:0	3.43	4.47	0.20	0.003
C16:0	32.44	33.69	0.68	0.218
C18:0	3.76	3.91	0.18	0.548
∑SFA^3^	39.63	42.07	0.73	0.035
C16:1 (n-7)	8.42	10.77	0.23	<0.001
C18:1 (n-9)	27.97	20.73	0.72	<0.001
∑MUFA^3^	36.40	31.50	0.72	<0.001
C18:2 (n-6)	13.56	10.78	0.44	<0.001
C20:4 (n-6)	0.39	0.29	0.03	0.008
∑(n-6) PUFA^3^	13.95	11.07	0.45	<0.001
C18:3 (n-3)	0.56	0.65	0.04	0.104
C20:5 (n-3)	0.04	2.71	0.07	<0.001
C22:5 (n-3)	0.10	1.12	0.04	<0.001
C22:6 (n-3)	0.07	2.53	0.06	<0.001
∑(n-3) PUFA^3^	0.77	7.00	0.11	<0.001
∑PUFA^3^	14.71	18.07	0.49	<0.001

^1^Values are means ± pooled SEM, control n = 9, treatment n = 6.

^2^Fatty acids are designated by the number of carbon atoms followed by the number of double bonds. The position of the first double bond relative to the methyl (n) end of the molecule is also indicated.

^3^SFA: saturated fatty acids, MUFA: monounsaturated fatty acids, PUFA: polyunsaturated fatty acids; ∑SFA: the sum of C14:0, C16:0, and C18:0; ∑MUFA: the sum of C16:1 (n-7) and C18:1 (n-9); ∑(n-6) PUFA: the sum of C18:2 (n-6) and C20:4 (n-6); ∑(n-3) PUFA: the sum of C18:3 (n-3), C20:5 (n-3), C22:5 (n-3), and C22:6 (n-3); ∑PUFA: the sum of ∑(n-6) PUFA and ∑(n-3) PUFA.

**Table 5:** Fatty acids composition of *longissimus dorsi *muscle.

Fatty acid	Diet^1^				SEM	*P*-value^2^		
	CC	CT	TC	TT		L	PW	L × PW
Fatty acid composition, g/100 g total fatty acid								
C14:0	1.77	1.72	1.33	1.58	0.10	0.03	0.34	0.17
C16:0	25.07	23.36	22.66	21.62	0.45	0.01	0.02	0.49
C16:1n-7	4.01	4.07	3.02	4.08	0.16	0.11	0.01	0.02
C18:0	10.24	10.78	10.87	7.38	0.59	0.03	0.06	0.01
C18:1n-9	33.41	26.10	34.05	31.53	1.26	0.06	0.01	0.11
C18:1n-7	2.97	3.87	3.50	3.56	0.24	0.66	0.06	0.10
C18:2n-6	16.09	17.38	15.90	13.76	0.52	0.06	0.44	0.02
C18:3n-3	0.65	0.76	0.64	0.79	0.04	0.77	0.01	0.61
C20:0	0.11	0.09	0.09	0.06	0.01	<0.01	<0.01	0.44
C20:1n-9	0.44	0.79	0.56	1.00	0.04	<0.01	<0.01	0.35
C20:4n-6	2.09	1.80	1.49	0.68	0.24	0.01	0.06	0.31
C20:5n-3	0.07	1.43	0.19	1.34	0.10	0.87	<0.01	0.38
C22:5n-3	0.10	0.29	0.84	0.83	0.04	0.01	<0.01	0.04
C22:6n-3	0.22	1.19	0.45	1.57	0.15	0.13	<0.01	0.65
SFA^3^	34.29	31.95	32.96	30.55	0.60	<0.01	<0.01	0.08
MUFA^3^	40.83	34.82	41.14	40.17	1.28	0.05	0.02	0.07
n-6 PUFA^3^	18.18	19.18	17.39	14.43	0.61	0.02	0.16	0.02
n-3 PUFA^3^	1.03	4.22	1.57	4.54	0.27	0.24	<0.01	0.78
PUFA^3^	19.21	23.40	18.96	18.97	0.71	0.08	0.03	0.03
n-6/n-3^3^	17.71	4.55	11.24	3.30	0.65	<0.01	<0.01	0.01
P/S^3^	0.56	0.65	0.54	0.57	0.03	0.13	0.10	0.34

^1^Values are means ± pooled SEM, *n* = 7. CC: all period fed lard diet; CT: post-weaning period fed fish oil diet; TC: lactation period fed fish oil diet; TT: all period fed fish oil diet.

^2^Effects of lactation (L) and the post-weaning (PW) or the interaction between lactation and the starter diet period (L × PW).

^3^Fatty acids are designated by the number of carbon atoms followed by the number of double bonds. The position of the first double bond relative to the methyl (n) end of the molecule is also indicated. SFA: saturated fatty acids, MUFA: monounsaturated fatty acids, PUFA: polyunsaturated fatty acids; ∑SFA: the sum of C14:0, C16:0, and C18:0; ∑MUFA: the sum of C16:1 (n-7) and C18:1 (n-9); ∑(n-6) PUFA: the sum of C18:2 (n-6) and C20:4 (n-6); *∑*(n-3) PUFA: the sum of C18:3 (n-3), C20:5 (n-3), C22:5 (n-3), and C22:6 (n-3); ∑PUFA: the sum of ∑(n-6) PUFA and ∑(n-3) PUFA.

**Table 6:** Effect of fish oil supplementation during late gestation and lactation on the performance of suckling piglets^1^.

Item	Diet		SEM	*P*-value
	C	T		
Litter weight at delivery, kg	15.41	15.72	0.62	0.723
Body weight at birth, kg	1.58	1.62	0.07	0.811
Body weight at 21 d, kg	5.91	6.35	0.15	0.080
Average daily gain, g	206	225	6	0.048

^1^Values are means ± pooled SEM, control *n* = 10, treatment *n* = 8.

**Table 7:** Growth performance of weaned piglets born to fish oil-treated and control sows and fed diets with and without fish oil supplementation in the post-weaning period.

Item	Diet^1^				SEM	*P*-value^2^		
	CC	CT	TC	TT		L	PW	L × PW
Body weight (35 d), kg	9.50	9.50	9.86	9.96	0.41		0.789	
Body weight (70 d), kg	19.66^ab^	17.91^b^	22.14^a^	20.45^ab^	1.20	0.047	0.163	0.979
Average daily gain, g	290^ab^	240^b^	351^a^	300^ab^	27	0.036	0.072	0.984
Average daily feed intake, g	533^ab^	493^b^	656^a^	574^ab^	44	0.028	0.179	0.631
Feed : gain	1.88	2.06	1.88	1.94	0.06	0.328	0.075	0.328

^1^Values are means ± pooled SEM, *n* = 7. CC: all period fed lard diet; CT: post-weaning period fed fish oil diet; TC: lactation period fed fish oil diet; TT: all period fed fish oil diet.

^2^Effects of lactation (L) and the post-weaning (PW) or the interaction between lactation and the starter diet period (L × PW).

^a,b^Means with different letters are significantly different (*P* < 0.05) among groups.

**Table 8:** The correlation coefficients between ADG and expressions of proinflammatory cytokine genes in *longissimus dorsi* muscle and spleen.

Item	*Longissimus dorsi* muscle			Spleen		
	*IL-1β* ^1^	*IL*-6^1^	*TNF*-*α* ^1^	*IL-1*β**	*IL-6 *	*TNF-*α**
ADG						
Correlation coefficients (r)	−0.7692	−0.3276	−0.3956	−0.7595	−0.3028	−0.2871
*P*-value	0.001	0.233	0.161	0.001	0.293	0.300

^1^ *IL-1*β**: interleukin 1*β*, *IL-6*: interleukin 6, *TNF-*α**: tumor necrosis factor-*α*.
